# Antitumor Effect and Induced Immune Response Following Exposure of Hexaminolevulinate and Blue Light in Combination with Checkpoint Inhibitor in an Orthotopic Model of Rat Bladder Cancer

**DOI:** 10.3390/biomedicines10030548

**Published:** 2022-02-25

**Authors:** Laureline Lamy, Jacques Thomas, Agnès Leroux, Jean-François Bisson, Kari Myren, Aslak Godal, Gry Stensrud, Lina Bezdetnaya

**Affiliations:** 1 Centre de Recherche en Automatique de Nancy, Centre National de la Recherche Scientifique, UMR 7039, Université de Lorraine, Campus Sciences, Boulevard des Aiguillette, 54506 Vandoeuvre-lès-Nancy, France; la.lamy@nancy.unicancer.fr; 2 Research Department, Institut de Cancérologie de Lorraine, 6 Avenue de Bourgogne, 54519 Vandoeuvre-lès-Nancy, France; 3 Service de Biopathologie, Institut de Cancérologie de Lorraine, 54506 Vandoeuvre-Lès-Nancy, France; j.thomas@nancy.unicancer.fr (J.T.); a.leroux@nancy.unicancer.fr (A.L.); 4 ETAP-Lab., 13 Rue du Bois de la Champelle, 54500 Vandoeuvre-les-Nancy, France; jfbisson@etap-lab.com; 5 Photocure ASA, Hoffsveien 4, 0275 Oslo, Norway; myren@oncoinvent.com (K.M.); asgodal@online.no (A.G.); gry.stensrud@lytixbiopharma.com (G.S.)

**Keywords:** bladder cancer, Hexvix^®^, photodiagnosis, PDT, immune checkpoints

## Abstract

Previous studies have found that use of hexaminolevulinate (HAL) and blue light cystoscopy (BLC) during treatment of bladder cancer had a positive impact on overall survival after later cystectomy, indicating a potential treatment effect beyond improved diagnostic accuracy. The aim of our study was to determine whether HAL and BL mimicking clinically relevant doses in an orthotopic rat model could have therapeutic effect by inducing modulation of a tumor-specific immune response. We also assessed whether administration with a checkpoint inhibitor could potentiate any effects observed. Rats were subjected to HAL BL alone and in combination with anti-PD-L1 and assessed for anti-tumor effects and effects on immune markers. Positive anti-tumor effect was observed in 63% and 31% of rats after, respectively, 12 and 30 days after the procedure, together with a localization effect of CD3+ and CD8+ cells after 30 days. Anti-tumor effect at 30 days increases from 31% up to 38% when combined with intravesical anti-PD-L1. In conclusion, our study demonstrated treatment effects with indications of systemic immune activation at diagnostic doses of HAL and blue light. The observed treatment effect seemed to be enhanced when used in combination with intravesically administrated immune checkpoint inhibitor.

## 1. Introduction

Urothelial carcinoma is the fifth most prevalent cancer worldwide, where non-muscle-invasive bladder cancer (NMIBC) constitutes 75% of primary diagnosis [1]. NMIBC is characterized by frequent recurrences and progression to muscle-invasive bladder cancer within 24 months after treatment with transurethral resection of bladder tumor (TURBT) followed by intravesical bacillus Calmette–Guérin (BCG) instillation [2].

Use of the photosensitizer hexaminolevulinate (HAL, Hexvix^®^) and blue light cystoscopy (BLC^®^) has been introduced to increase detection of tumors during diagnosis and surgical treatment of bladder cancer [3]. HAL induces preferential accumulation of intracellular protoporphyrin IX (PpIX) in neoplastic tissue. Illumination with blue light (BL; 380–440 nm) produces a clearly demarcated red fluorescence from malignant tissue. Increased detection leads to more complete tumor resection, resulting in improved short and sustained long-term recurrence rates [4,5]. BLC with HAL is recognized and recommended across international and national guidelines [6,7].

Whereas photodynamic diagnosis (PDD) is a diagnostic modality, photodynamic therapy (PDT) is a promising technology in the treatment of various cancer types. PDT combines the administration of a photosensitizer with the illumination of light of specific wavelength to generate cytotoxic singlet oxygen. PDT destroys tumor cells via direct cell destruction and indirectly via vascular shutdown and induction of acute local inflammatory response resulting in immune system activation [8,9,10,11,12]. HAL-mediated PDT with WL was earlier tested with curative purpose in patients with urothelial carcinoma after TUR and demonstrated efficacy of the treatment without major side effects [13]. Intriguingly, two studies have demonstrated positive impact on patient outcomes after cystectomy in patients who had undergone BLC prior to their cystectomy compared to WL-TURB, indicating an additional effect of BLC beyond pure detection [14,15]. Both overall survival and cancer-specific survival were significantly higher in the BLC group, and prior BLC with HAL was found to be an independent predictor of survival after radical cystectomy [15].

A promising new age of immunotherapy has arrived in the form of checkpoint inhibition [16]. Tumor cells can escape immune surveillance by upregulating PD-L1/PD-1 expression in cells of tumor microenvironment. The anti-PD-1 and anti-PD-L1 antibodies bind, respectively, to PD-1 on T cells and PD-L1 on cancer cells preventing the interaction of PD-1 and PD-L1, thus reactivating the anti-tumor immune response of cytotoxic T cells [17]. For bladder cancer treatment, five such drugs have been approved by the FDA for use in different settings, including pembrolizumab for BCG-unresponsive high-risk NMIBC [18,19]. Immune checkpoint inhibitors have demonstrated a higher benefit in heavy CD8+ infiltrated tumors and in tumors with high tumor mutational burden [19]. The role of checkpoint inhibition in NMIBC has been summarized by Hahn and coworkers [20].

Several studies on the combined effect of PDT with photosensitizers and immune checkpoint inhibition in preclinical models aiming to improve the therapeutic efficiency have been reported [21]. Enhanced anti-tumor efficacy was demonstrated in murine tumor models including breast, subcutaneous, melanoma, renal cell carcinoma, and colon through a combined action of checkpoint inhibitors and PDT. The local treatment of tumor together with the systemic administration of checkpoint inhibitors primed an immune response resulting in increased infiltration of activated CD8+ T cells in the primary tumor, but also in secondary tumors/metastases indicating an abscopal effect. Moreover, an immunological memory effect was developed as PDT-treated tumor-free mice were protected against developing new tumors when re-challenged [21,22,23,24,25].

We hypothesized that the positive impact on patient outcomes in patients who had undergone BLC prior to cystectomy could be caused by a direct anti-tumor effect and/or activation of the immune system as seen with PDT. Therefore, as a proof-of principle study for future translation study, we investigated whether intravesical administration of HAL followed by a diagnostic blue light illumination regime (PDD) could have an anti- tumor and immune modulating effects, as well as increasing susceptibility to PD-1/PD-L1 pathway inhibition in a preclinical model. Rats were therefore treated with HAL and BL in an orthotopic model of bladder cancer and subjected to histopathological analysis and assessment of immune markers. Co-administration of HAL BL with a checkpoint inhibitor was further tested in this model aiming to assess for potentiation of anti-tumor effects.

## 2. Materials and Methods

### 2.1. Tumor Cells Culture

The rat bladder TCC cell line AY-27 has been established as a primary bladder tumor in Fischer 344 rats by feeding the rats with N-(4-[5-nitro-2-furyl]-2-thiazolyl) formamide. The bladder tumor cells were cultured in vitro as a monolayer at 37 °C in a humidified 5% CO_2_ and 95% air atmosphere in RPMI 1640 culture media (Sigma, Saint-Quentin Fallavier, France) complemented with 9% FCS, 1% L-glutamine and 1% antibiotic/antimycotic solution. Cells were passaged when nearly confluent. The cell culture medium and other culture ingredients and PBS were obtained from Sigma (France).

### 2.2. Orthotopic Tumor Model

Nine-week-old female Fischer rats (Charles River Laboratories, Chatillon-sur-Chalronne France) weighing 140–165 g were housed in groups of 4 rats per cage in a room with 12-h inverted light/dark cycle and controlled temperature (22 ± 2 °C). Animal care protocols were used in accordance with the guidelines of the European Communities Council Directive on the approximation of laws, regulations, and administrative provisions of the Member States regarding the protection of animals used for scientific purposes, the National Institutes of Health Guide for the care and use of laboratory animals, and the ASAB Ethical Committee. The studies received approval from the French Ministry of Higher Education and Research (agreements no. APAFIS#14510 on 9 July 2018, and no. APAFIS#23597 on 3 March 2020). Animal supervision was performed daily. All efforts were made to prevent animal suffering.

Tumors were induced as initially described by Xiao et al. [26]. Briefly, animals were anaesthetized with an intraperitoneal injection (i.p.) of ketamine/xylazine mixture (54/6 mg·kg^−1^) to maintain ~1.5 h of anesthesia and fixed on animal boards kept at 37 °C. The anesthesia was completed by i.p. injection of 0.01 mg/kg opioid-based buprenorphine. After urethral catheterization of the bladder with a 16-gauge plastic intravenous cannula, the bladder urothelium was first conditioned with 0.5 mL HCl (0.1 N) for 15 s, neutralized with 0.5 mL (0.1 N) NaOH for 15 s, and immediately washed several times with sterile physiological serum. Then, a suspension of AY-27 cells (106 cells) in 0.5 mL of medium was instilled into the bladder via the catheter for 1 h.

### 2.3. Hexylaminolevulinate Preparation and Administration

Hexylaminolevulinate (HAL HCl (Mw 251)) was kindly provided by Photocure ASA, Oslo, Norway. HAL was dissolved in RPMI medium (without serum) immediately before instillation and 0.5 mL 8 mM were instilled intravesically and kept for 1 h. After bladder evacuation, bladders were washed three times with the PBS solution followed by illumination.

### 2.4. Illumination of Rat Bladders

Whole bladder illumination with blue light (BL) was performed at day 5 after tumor cell implantation using a 200 mW Modulight laser model ML 6500 with excitation wavelength at 405 nm, coupled to fiber with a cylindrical diffuser (1 × 5 mm, Medlight) placed in a central position in the bladder filled with 0.5 mL PBS. During illumination the irradiance was fixed at 7 mW/cm^2^ and a total light dose was 7.5 J/cm^2^. The illumination parameters were selected to mimic the irradiance and light doses used during a clinical blue light cystoscopy. The selected parameters were in the same range as reported by Karl Storz for their D-Light C Photodynamic Diagnosis (PDD) system [27].

### 2.5. Combination Therapy

**Figure 1:**
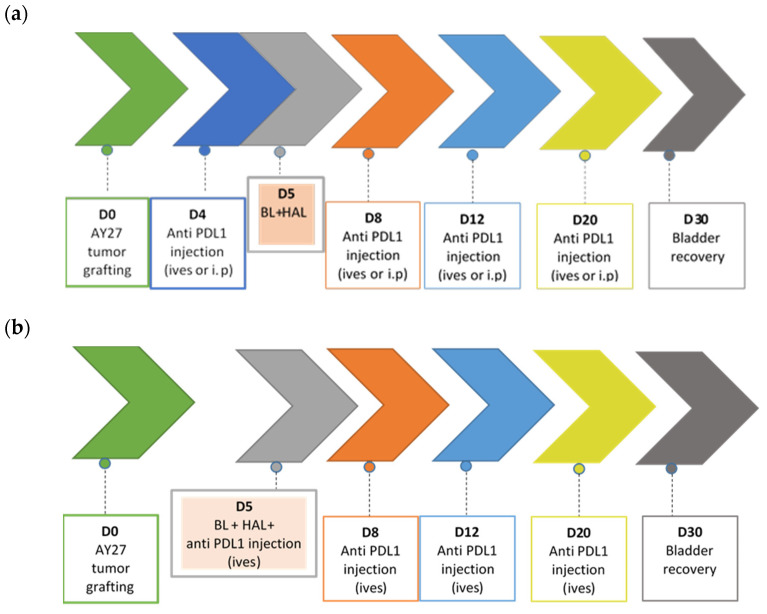
Experimental scheme of HAL BL treatment alone and in combination with anti PD-L1 injections. (**a**) Intraperitoneal (i.p.) and intravesical (ives) administrations; (**b**) anti PD-L1 co-administration with HAL (same syringe).

Mouse PD-L1 monoclonal antibody (clone 10F.9G2; Bio-XCell) was delivered by manufacturer as a liquid stock solution (7.50 mg/mL) and stored at 4 °C. Immunotherapy was conducted using two administration routes of anti-PD-L1: intravesical (ives) and intraperitoneal (i.p.). Treatment scheme was selected based on prior studies in rodents of combined action of immune checkpoints inhibitors with either radiotherapy or PDT [22,28]. Treatment scheme is presented in Figure 1.

#### 2.5.1. Intraperitoneal Injections (i.p.)

For i.p. injections, no additional anesthesia was required. Stock PD-L1 solution was diluted in endotoxin-free PBS immediately before use. At each of four PD-L1 injections, rats received 100 µL/rat of anti-mouse PD-L1 antibody at the concentration of 2 mg/mL. A treatment dose of 0.2 mg/rat/injection was selected based on prior published experience [28]. Injections were performed at days 4, 8, 12, and 20 after tumor implantation (Figure 1).

#### 2.5.2. Intravesical Instillations (ives)

Instillation was done during 1 h under ketamine/xylazine anesthesia. At each of four PD-L1 ives instillations, rats received 500 µL/rat of antibody at the concentration 7.5 mg/mL. The dose of 3.75 mg/rat/injection was selected to represent the dose used in clinical settings [29], recalculated for equivalent doses in rats. Two schedules of anti-PD-L1 ives instillations were used: on days 4, 8, 12, and 20 or on days 5, 8, 12, and 20 after tumor cells implantation. In the second schedule, the first anti-PDL1 instillation (on day 5) was co-administered together with HAL (in one syringe) (Figure 1).

### 2.6. Pathological and Immunological Analysis

The rats were sacrificed by intracardiac overdose of pentobarbital at 7, 12, and 30 days after tumor cell inoculation for pathological and immunological analysis.

#### 2.6.1. Hematoxylin-Eosin-Safran (HES) Analysis

Cystectomy specimens were fixed with 4% formaldehyde before paraffin embedding for about 4 h. Afterwards, the bladders were cut in four parts and placed in cassette. After approximately 48 h in dehydratation bath, the bladders were paraffined. Two sections (5 µm) obtained serially at 0.2 mm intervals were stained with hematoxylin-Eosin-Safran (automatic methods).

**Figure 2:**
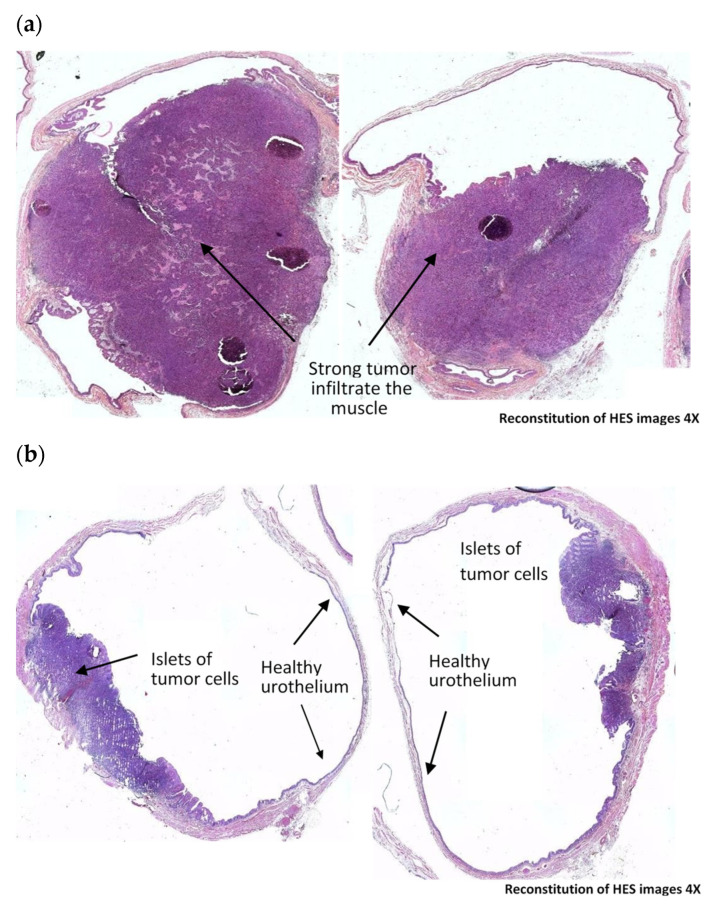

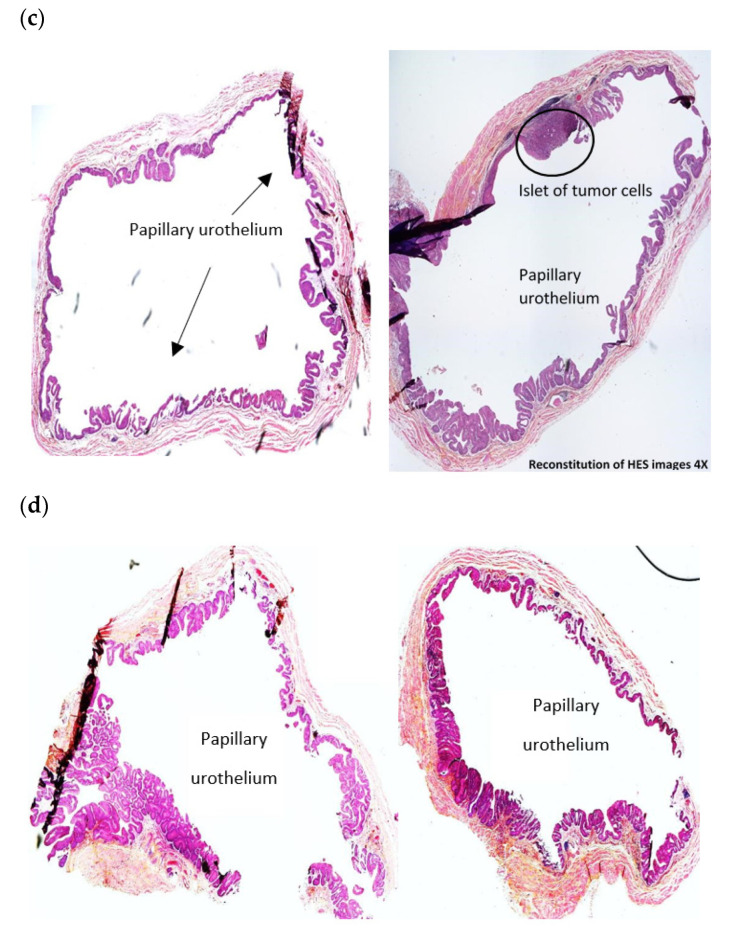
Tumor response grades (adapted with modification from [30]) and bladder tumor typical images corresponding to different response rates. (**a**) No response (NR): bladders with strong muscle-infiltrative tumor; (**b**) Moderate response (MR): bladders with few tumor cells or islets of tumor cells in several areas; (**c**) Near Complete Response (Near CR); (**d**) Complete Response (CR).

Therapeutic efficacy in rat bladders was graded into 4 groups (Table 1), based on modified Dworak TGR (tumor regression rate) system [30]. These four groups stand for No Response (NR), Moderate Response (MR), Near Complete Response (near CR) and Complete Response (CR). Typical images corresponding to each of four grades are depicted in Figure 2. Positive anti-tumor effect was considered as a sum of near CR and CR.

#### 2.6.2. Assessment of Immune Cells in Tissues

Inflammatory cells were evaluated by CD3+, CD8+, and CD4+ lymphocytes labeling. CD8+ and CD4+ antibodies staining protocol was adapted from a protocol published by Bressenot [31] with slight modifications. CD4+ lymphocytes labeling was performed according to manufacturer recommendations. CD8+ and CD4+ immunohistochemistry (IHC) was carried out on 4 µm thick deparaffinized sections in a manual mode. Before staining, the sections were subjected to heat-induced epitope retrieval by incubation in 0.01 M Tris/EDTA solution (pH 8) at 95 °C for 40 min, followed by 10 min of cooling. Slices were briefly rinsed with water. The sample on the slide was spotted with dakopen. PBS Tween (0.1 M phosphate buffer, pH 7.4, 0.1% (*v*/*v*) Tween 20) was added to each spotted sample for 30 min. Endogenous peroxydase activity at each spot was blocked by 5 min incubation in a 3% hydrogen peroxide solution in distilled water. Primary antibody was diluted in 1% (*m*/*v*) BSA. With this aim, purified mouse anti-RatCD8a (1: 100 diluted, BD Pharmingen) was incubated at room temperature for 1 h and mouse anti-rat CD4 W3/25 (1: 200 diluted, Bio-Rad, Marnes-La- Coquette, France) was incubated at 4 °C overnight. After that, the slices were washed twice with PBST, each for 10 min and incubated with secondary antibody, biotinylated goat anti-mouse Ig (multiple adsorption), diluted 1:100 for CD8, and biotinylated donkey anti-mouse IgG, diluted 1:50 for CD4. Secondary antibody remained on the slides for 1 h at room temperature.

Afterwards, the slices were washed twice with PBST, each for 10 min, followed by incubation with streptavidin-peroxidase (BD Pharmingen) for 30 min at room temperature. The slices were washed twice with PBST (each for 5 min). The Novared TR system (Abcys, Paris, France) was used to detect bound peroxidase (12 min). Nuclear counterstaining was performed with twice diluted Harris hematoxylin (1 min). At the end of the procedure, the slices were dehydrated in three baths of alcohol (95/100/xylen) and covered by lamellas. IHC CD3 was carried out on 4µm thick deparaffinized sections and further staining was performed with automated immunostainer (BenchMark ULTRA instrument) with flex rabbit polyclonal anti-CD3+ human antibody (Agilent) as primary antibody.

Inflammation was assessed by the expression of CD8+, CD4+ and CD3+ lymphocytes. The ratio of all cell nuclei (Olympus, X40) at several images (4 or 5) achieving totally about 1000 cells to CD8+, CD4+, and CD3+ lymphocytes was calculated.

#### 2.6.3. PD-L1 Expression in Tumor Cells

PD-L1 expression was assessed according to manufacturer recommendations. PD-L1 protein detection in paraffin-embedded bladders was determined with mouse monoclonal anti-PD-L1 antibody, Clone 22C3 against PD-L1 (Agilent). Staining was performed by using an automated immunostainer (BenchMark ULTRA instrument). The OptiView DAB IHC Detection Kit (OptiView) is an indirect, biotin-free system for detecting rabbit primary antibody. After this labeling, the slides were visualized by white light microscopy. In the first lieu, assessment at low magnification was performed to identify homogeneity of staining. Afterwards, at high magnification, the areas of interest were analyzed to separate immune PD-L1 positives cells from tumor cells. Finally, PD-L1 positive tumor cells were quantified.

**Figure 3:**
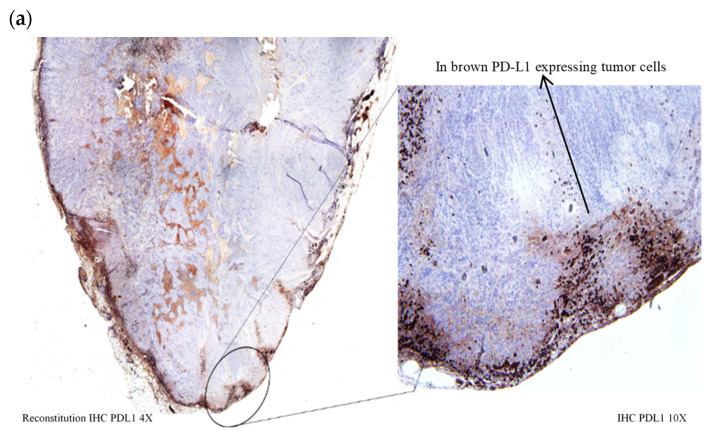

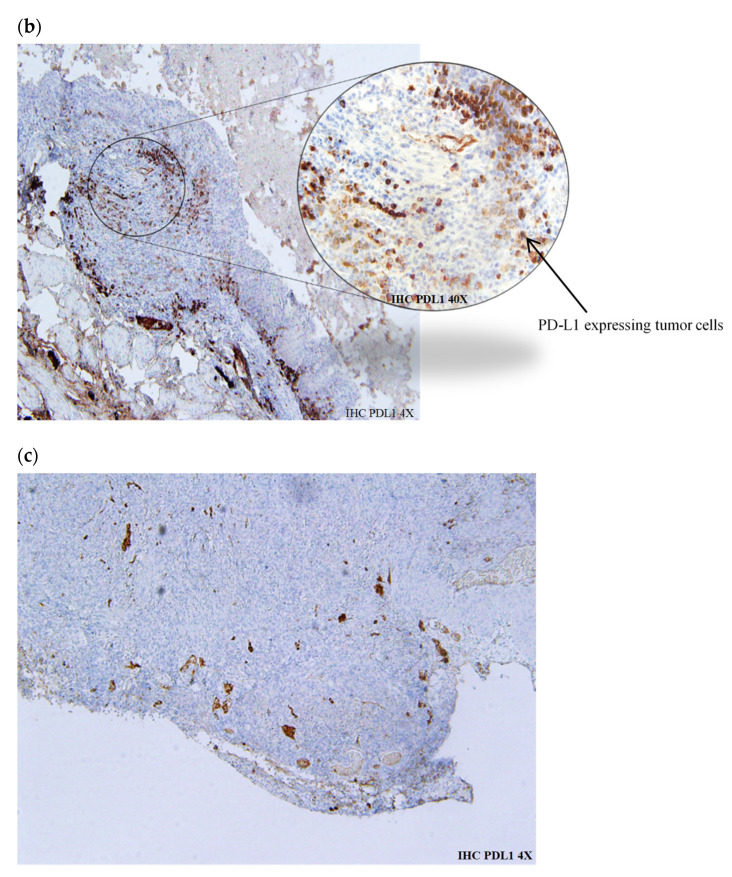
Representative images of PD-L1 staining. (**a**) Typical image of strong membranous PD-L1 expression (>50%). Example of bladder sample treated with HAL BL. Membranous PD-L1 expression in tumor cells (10×). Photo captured at 12 days post-grafting; (**b**) typical image of moderate membranous PD-L1 expression (1 < PD-L1 < 49%). Example of bladder sample treated with BL. Membranous PD-L1 expression in tumor cells (×4). Photo captured at 30 days post-grafting; (**c**) typical image of weak membranous PD-L1 expression (<1%). Example of bladder sample treated with HAL BL (×4). Photo captured at 30 days post-grafting.

PD-L1 expression was evaluated according to Percentage Staining (PS) of tumor cells used in conventional clinical practice. PS was graded into strong (50 < PD-L1 < 100%); moderate (1 < PD-L1 < 49%) and weak expression (PD-L1 < 1%). Typical images corresponding to different levels of PD-L1 expression are indicated in Figure 3.

### 2.7. Statistical Analysis

The overall number of animals used in this study was 85. The number of rats differed in each group. For the HES-assessed anti-tumor effect, the numbers of rats in each group were between 6 and 13. For the IHC-assessed PD-L1 expression, the numbers of rats in each group were from 4 to 12.

Expression of T lymphocytes is presented as mean ± SEM; comparison between groups was performed using non-parametric Mann–Whitney U test. *p* values < 0.05 were considered statistically significant.

The correlation between variables and responses was determined by partial least squares regression. The significant main, interaction, and squared terms were found by the backward selection procedure removing one-by-one of the most insignificant terms until only significant terms remained (*p* < 0.05). The statistical calculations were performed by the statistical software Modde Pro version 12.0.1, Sartorius Stedim data analytics AB.

## 3. Results

### 3.1. HAL-PDD-Mediated Therapeutic Effect in AY27 Orthotopic Bladder Tumors

We studied the therapeutic effect of HAL and BL illumination at different time points after tumor cell inoculation. Intravesical instillation of HAL and BL illumination was performed five days after tumor grafting. A significant positive therapeutic outcome (63%; *p* < 0.05) was demonstrated when the rats subjected to HAL BL were assessed 12 days after tumor grafting (n = 8) (Table 2). Five rats were either tumor-free (CR) or with few tumor cells (Near CR), while two rats responded moderately (MR). In the last case, we observed a tumor confined to bladder wall but without entire wall invasion. In one rat the treatment was ineffective (NR) (Table 2).

The positive therapeutic outcome was not sustained in rats sacrificed 30 days post-grafting where the beneficial effect was significantly reduced to 31% (n = 13; *p* < 0.05). After 30 days, four out of 13 rats were tumor-free (CR), one rat responded moderately (MR), but 8 out of 13 bladders demonstrated strong muscle-infiltrative tumors (NR) (Table 2). Two control groups were also assessed 30 days after tumor grafting. In the untreated control tumor group (CTR ND NL; n = 12) HES demonstrated muscle invasive tumors in the chorion in all rats (Table 2). In control rats subjected to BL illumination only (CTR BL; n = 6), the bladders of all six rats were heavily invaded with tumor (Table 2). All rats in all treatment group survived without any visible sign of suffering.

### 3.2. Assessment of Immunological Markers

In all treated groups we observed a strong inflammation, and therefore in the next step we assessed the recruitment of lymphocytes. The CD3+ inflammatory marker and two subsets of T cells, namely, CD4+ T helper cells and CD8+ cytotoxic T lymphocytes were quantified and their localization was assessed in extracted tumors at different times after tumor grafting.

**Figure 4:**
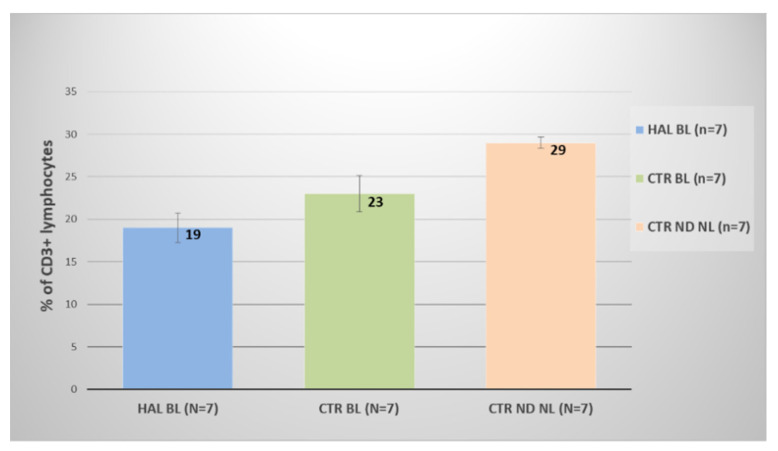
Expression of CD3+ lymphocytes in rat bladder tumors 30 days after tumor grafting in control and experimental groups.

Expression of CD3+ in untreated control tumors (CTR ND NL) at 12 days post grafting was 17% (data not shown) and increased significantly to 29% at 30 days post tumor grafting (*p* < 0.01) (Figure 4). This increase in CD3+ expression in untreated tumors confirms a chronic inflammatory status inherent to tumor presence. CD3+ expression in rat bladders subjected to HAL BL was significantly lower (19%; *p* < 0.05) than that in non-treated group (CTR ND NL) but not different from the control BL group (23%) (Figure 4).

CD8+ expression was not significantly different between tested groups 30 days after tumor grafting and varied between 18% and 21% (data not shown). The expression of CD4+ was overall lower than that of CD8+ and varied between 7% and 11% but was not significantly different between tested groups (*p* > 0.1) (data not shown).

**Figure 5:**
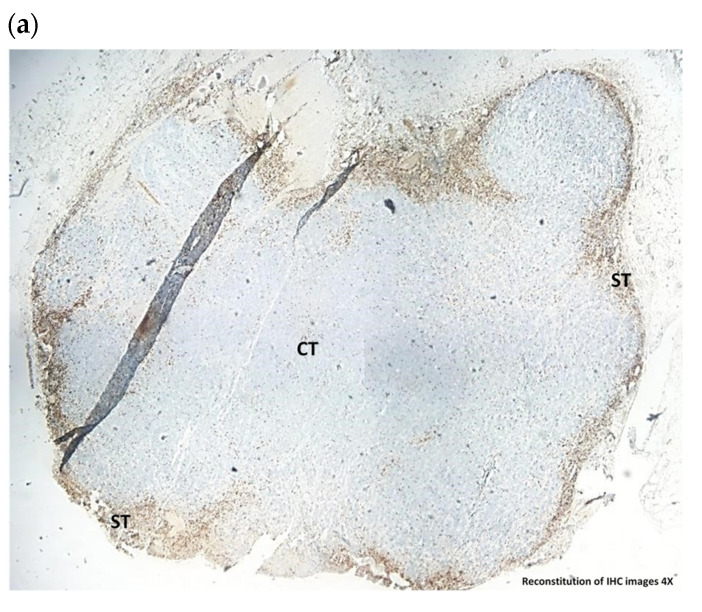

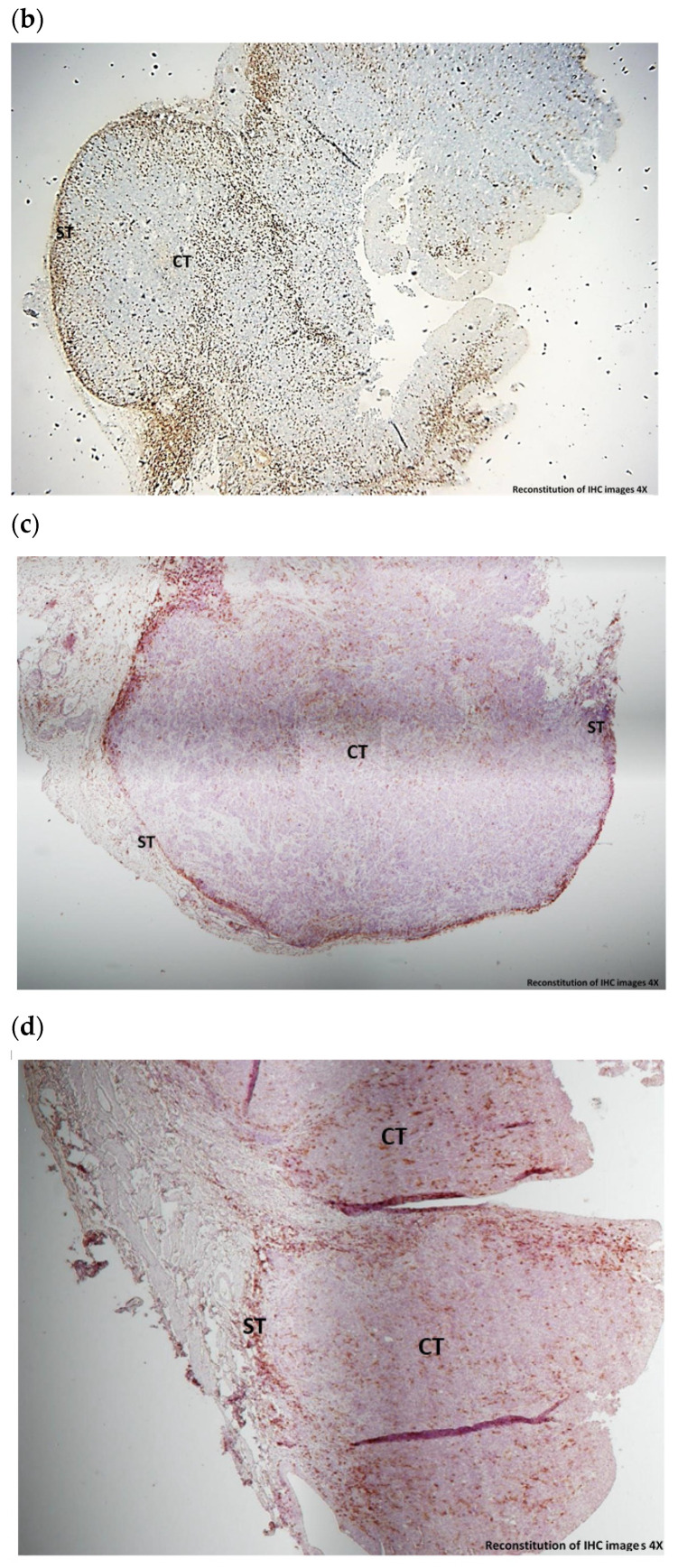
Typical images of CD3+ lymphocytes (**a**,**b**) and CD8+ lymphocytes (**c**,**d**) in bladder tumors 30 days post-grafting. (**a**) CD3+ lymphocytes in CTR BL sample displaying peripheral localization in stroma regions (ST) (×4); (**b**) CD3+ lymphocytes in HAL BL sample displaying localization around tumor cells in the center of tumor (CT) (×4); (**c**) CD8+ lymphocytes in CTR BL sample displaying peripheral localization in stroma regions (ST) (×10); (**d**) CD8+ lymphocytes in HAL BL sample displaying localization around tumor cells in the center of tumor (CT) (×10).

Albeit no quantitative difference was observed in the expression of CD3+ after HAL BL compared to BL only, close examination of the slides revealed a different localization of the CD3+ lymphocytes. A strong CD3+ localization around tumor cells was observed in the HAL BL group, whereas in the CTR BL group, CD3+ lymphocytes were mostly localized on the periphery of tumor sample (Figure 5a,b). The same distribution pattern was observed for CD8+ lymphocytes (Figure 5c,d), but unlike CD3+ cells, partial localization was also seen in the control BL group. CD4+ cells were distributed on the periphery in both experimental groups (data not shown). Periphery localization of CD3+, CD4+ and CD8+ was also observed for untreated control tumors (data not shown). The localization of CD3+ and CD8+ lymphocytes around tumor cells in HAL BL-treated rat bladders could indicate a stimulation of the immune system explaining the anti-tumor effect (Table 2). Therefore, in the next step we studied whether the anti-tumor effect could be improved by combining exposure of HAL and BL with anti-PD-L1 immunotherapy.

### 3.3. IHC-Assessed PD-L1 Expression in Bladder Tumor Cells

PD-L1 expression was assessed in tumor cells collected from rats exposed to HAL BL, BL only and untreated control tumor at 7 days, 12 days, and 30 days after tumor grafting (Table 3). Only weak/moderate expression of PD-L1 was seen in the HAL BL and CTR BL groups at 7 days after tumor grafting (Table 3). Twelve days after tumor grafting, a transient increase to strong PD-L1 expression was observed in all experimental groups (Table 3; Figure 3a) but did not sustain. Indeed, 30 days after tumor grafting the PD-L1 expression returned back to weak/moderate levels (Table 3; Figure 3b,c).

### 3.4. Therapeutic Effect of Immune Checkpoint Therapy in AY27 Orthotopic Bladder Tumors

The increased PD-L1 expression 12 days after tumor grafting justified further experiments with anti-PD-L1 immunotherapy. Two different administration routes of anti-PD-L1 were performed: intravesical (ives) and intraperitoneal (i.p.) and two different dosing regimens (Figure 1). No anti-tumor effect was observed in the controls treated with anti-PD-L1 ives or i.p. alone 30 days after tumor grafting (Table 4). However, all groups exposed to HAL showed a positive anti-tumor effect. This effect was significant for rats exposed to HAL BL alone and in combination with anti-PDL-1 immunotherapy compared with control groups (*p* < 0.05). A treatment regime with intravesical co-administration of HAL BL and anti-PD-L1 seemed to be more advantageous (38%) compared to the other groups, although the difference was not statistically significant (*p* > 0.05) (Table 4). It is important to note that no signs of suffering were noted with the combination with checkpoints inhibitors.

## 4. Discussion

In our study, we used blue light illumination after intravesical instillation of hexylaminolevulinate alone and in combination with checkpoint inhibitor in rat bladders with orthotopic tumors. This tumor model mimics bladder cancer in humans standing mostly for NMIBC, with a progression to MIBC (stages II-III) [26,32,33]. Studies of photodynamic therapy (PDT) with HAL and red light has been performed in this orthotopic bladder cancer model, demonstrating a positive short-term (48 h–168 h) therapeutic effect [34,35]. Therefore, this model should be suitable for our experiments.

In terms of dosimetry, the idea was to mimic the light doses used during a clinical blue light cystoscopy (PhotoDynamic Diagnosis, PDD). Based on measurements of blue light intensity from the tip of the cystoscope, the average range of light intensities was calculated at the distance between the scope and tumor during a PDD bladder investigation assuming a working distance between 3 and 5 cm. Furthermore, the age of the lamp was considered since its intensity varies with lamp age between 100–60% (lower limit) of full intensity. Based on the above, a range of irradiances of 1.8–12.3 mW/cm^2^ (mean 7.0 mW/cm^2^) was obtained. In our case, the delivered light doses were calculated by multiplying the above range with the range of typical blue light exposure times during tumor-resection (2–20 min) yielding a range of light doses of 0.2–14.8 J/cm^2^ (mean 7.5 J/cm^2^). Precise light dosing in in vivo studies, especially in hollow organs, is difficult to conduct due to variations in multiply parameters e.g., light diffusion, light scattering, and exact tumor localization. Therefore, certain caution is needed while interpreting the results. Based on these calculations, we used a fixed irradiance at 7 mW/cm^2^ and a total light dose of 7.5 J/cm^2^. This is in the same range as documented by Karl Storz for their D-Light C Photodynamic Diagnosis (PDD) system [27]. They report irradiance of blue light (mW/cm^2^) of 1.3, 3.5, and 32 for distances from tissue of 5, 3, and 1 cm, respectively.

In this study, we found a significant anti-tumor effect at observation end point, which was fixed at 30 days after tumor grafting with complete response (CR) in 31% (4/13 rats) of animals exposed to HAL and BL (Table 2). At the same time, we observed a strong tumor infiltration with CD3+ and CD8+ T cells in this experimental group indicating a T cell-mediated immune response. Enhancement of T cell activation has also been reported in studies of HAL red light PDT in a similar orthotopic bladder cancer model in rats [32]. The T cells infiltration is a result of an alteration in the tumor microenvironment, stimulating the release of different mediators and recruiting lymphocytes from the circulating blood to the tumor [36,37]. It appears that exposure of HAL BL as used during a diagnostic PDD procedure may result in a similar immune stimulation with activating of the innate and adaptive pathways [9,11,12]. It is likely that this immune system activation is the reason for the anti-tumor effect in the rats exposed to HAL and BL. A higher proportion of rats showed an anti-tumor effect 12 days after being exposed to HAL BL. At this time point, 63% of the rats showed CR (2/8 rats) or near CR (3/8) (Table 2). During the time period between 12 and 30 days, T cell dysfunction, exhaustion, and tolerance may happen due to the ligation of PD-1 with PD-L1 ligand, thus contributing to diminishing of HAL BL long-term effect. PD-1 or PD-L1 blockade can lead to an enhancement of anti-tumor activity [20]. It is also possible that the immune stimulation following single exposure to HAL and BL is not strong enough to control the tumor growth in this model. With the aim of achieving a sustained anti-tumor effect and to assess whether there could be an additional treatment effect we applied a combination of HAL and BL with targeted anti-PD-1 immunotherapy. PD-L1 is strongly expressed in MIBC but also in advanced stages of NMIBC [28]. Two administration routes of anti-PD-1 were tested in this model, intravesical and intraperitoneal. Irrespective of intravesical or intraperitoneal administration, targeted immunotherapy administered alone was ineffective as none of the tested rats responded positively (Table 4). Contrary, all groups exposed to HAL showed a positive anti-tumor effect although this effect was statistically significant only for rats exposed to HAL BL and where HAL BL was combined with intravesical administered anti-PDL-1 immunonotherapy. A treatment regime where HAL BL and anti-PD-L1 were co-administered intravesically at the same day provided the best antitumor effect where 38% of the rats showed near CR and CR. The somewhat lower anti-tumor effect when anti-PD-1 was administered i.p. (22%) compared to ives (30%) might also be explained by the lower concentration of anti-PD-1 used for this administration route.

The rather modest incremental effect seen when combining HAL BL with anti-PD-1 might be explained by the weak PD-L1 expression on the tumor cells. In our study, IHC-assessed PD-L1 expression in bladder cancer samples clearly showed that a strong expression of PD-L1 was evident 12 days after tumor implantation but it did not sustain (Table 3). Although expression of PD-L1 in tumors is correlated with higher response to therapy, this is not definite, and it is argued that an absence of PD-L1 expression in biopsies does not preclude response to anti-PD-1/PD-L1 immunotherapy [22]. It has earlier been reported that radiotherapy upregulates the PD-L1 expression on tumors cells, justifying the combination with anti-PD1/anti-PD-L1 inhibitors [28]. Moreover, CD8+ T cells are shown to be vital for the local and systemic therapeutic effects seen after combining radiation with checkpoint inhibitors [28]. As tumor infiltration with CD8+ T cells was not observed in our study until 30 days after tumor grafting, a potential upregulation of the PD-L1 expression as a consequence of T cell tumor infiltration amplifying the effect of the anti-PD-1 checkpoint inhibitor was not possible to rule out at observation end point for our study.

A recent study of Kirschner et al. compared efficacy of intravesical versus systemic (intraperitoneal) administration of an anti-PD-1 inhibitor in the treatment of localized bladder cancer in an orthotopic mouse model [38]. Intravesical anti-PD-1 administration had trend to improved survival thus providing effective anti-tumor treatment for bladder tumors. Further, increased CD8+ T cells infiltration in tumors was observed, especially after intravesical administration. Our study also demonstrated that intravesical administration of anti-PD-L1 combined with HAL BL has an improved survival over systemic i.p. administration, probably due to a higher local drug concentration after ives administration. Local immunotherapy administration into the bladder could represent a substantial clinical benefit, reducing systemic exposure and side effects. Our study in rats and the study reported by Kirschner et al. [38] in mice are encouraging in this respect. However, additional clinical trials are warranted before intravesical instillation of anti-PD-L1 could be widely used in humans alone or in combination with HAL and BL.

This is to the best of our knowledge the first proof-of principle study demonstrating an anti-tumor effect and indication of immune activation following HAL and BL exposure in vivo. Our hypothesis of immune activation could be strengthened by performing T cell depletion using anti-CD3. Moreover, IHC assessments could be supplemented by flow cytometry to give more conclusive results. Future studies can benefit from analysis of other immune markers including cytokine panels, antigen-specific CD8+, and granzyme B production.

## 5. Conclusions

We have demonstrated an anti-tumor effect of HAL and blue light when trying to mimic the dosing regimen of a photodynamic diagnostic procedure in an orthotropic bladder cancer model in rats. The anti-tumor effect is most probably pertaining to stimulation of the immune system as evident by tumor infiltration of CD3+ and CD8 + T cells. These results support our hypothesis that the positive impact on patient outcomes observed in patients who had undergone BLC prior to cystectomy could be explained by systemic immune activation induced by HAL and blue light. Combination of HAL and blue light with intravesical anti-PD-L1 resulted in increased anti-tumor effects. Further studies are warranted to explore the long-term effects of HAL and blue light alone or in combination with checkpoint inhibitors which should extend to investigate any systemic (abscopal) effects. The idea that local treatment with HAL and blue light can prime an immune response with potential additional effect of checkpoint inhibitors is also intriguing.

## Figures and Tables

**Table 1:** Tumor response grades (adapted with modification from [30]).

No Response (NR)	Moderate Response (MR)	Near Complete Response (Near CR)	Complete Response (CR)
Rat with strong muscle-infiltrative tumor	Rats with few tumor cells or islets of tumor cells in several areas	Rats with singular islet of tumor cells or very few tumor cells	Absence of tumor cells

**Table 2:** HES assessed therapeutic efficacy in rat bladders at 7, 12, and 30 days after tumor inoculation in control and experimental groups.

Time after Grafting (d)	Treatments	Tumor Response Grades					
		NR	MR	Near CR	CR	Near CR + CR	
12	HAL BL (n = 8)	1/8	2/8	3/8	2/8	5/8	63%
30	CTR ND NL (n = 12)	12/12	0/12	0/12	0/12	0/12	0%
	CTR BL (n = 6)	6/6	0/6	0/6	0/6	0/6	0%
	HAL BL (n = 13)	8/13	1/13	0/13	4/13	4/13	31%

HAL BL: HAL and blue light, CTR ND NL: control no drug no light, CTR BL: control blue light only, NR: no response, MR; moderate response, CR; complete response.

**Table 3:** IHC-assessed PD-L1 expression at 7, 12, and 30 days after tumor inoculation in control (CTR ND NL, CTR BL) and HAL BL-treated bladder tumors.

Time after Grafting (Days)	Treatments	50 < PD-L1 < 100% Strong	1 < PD-L1 < 49% Moderate	PD-L1 < 1% Weak
7	HAL BL (n = 4)	0/4	1/4	3/4
12	CTR ND NL (n = 4)	3/4	1/4	0/4
	CTR BL (n = 4)	3/4	1/4	0/4
	HAL BL (n = 6)	4/6	0/6	2/6
30	CTR ND NL (n = 11)	2/11	1/11	8/11
	CTR BL (n = 5)	2/5	2/5	1/5
	HAL BL (n = 12)	0/12	1/12	11/12

**Table 4:** HES-assessed anti-tumor effect in rat bladders 30 days after tumor inoculation. Tumor bearing rats were subjected to ives (intravesical) or i.p. (intraperitoneal) anti- PD-L1 immunotherapy.

Treatments	Tumor Response Grades					
	NR	MR	Near CR	CR	Near CR + CR	
CTR PDL1 ives (n = 9)	9/9	0/9	0/9	0/9	0/9	0%
CTR PDL1 i.p (n = 10)	10/10	0/10	0/10	0/10	0/10	0%
HAL BL PDL1 i.p (n = 9)	7/9	0/9	1/9	1/9	2/9	22%
HAL BL PDL1 ives (n = 10)	6/10	1/10	0/10	3/10	3/10	30%
HAL BL PD-L1 ives co-administered (n = 8)	3/8	2/8	2/8	1/8	3/8	38%
HAL BL * (n = 13)	8/13	1/13	0/13	4/13	4/13	31%

HAL BL: HAL and blue light, CTR: control no drug no light, NR: no response, MR; moderate response, CR; complete response. * Taken from Table 2.

## Data Availability

The dataset generated and analyzed in this study is not publicly available but may be obtained from the corresponding author upon reasonable request.
